# Self-reported Use of K2/Spice for Appetite Stimulation: A Case Report

**DOI:** 10.7759/cureus.7860

**Published:** 2020-04-27

**Authors:** Christopher Torres, Eduardo D Espiridion

**Affiliations:** 1 Psychiatry, Reading Hospital - Tower Health, West Reading, USA; 2 Psychiatry, Drexel University College of Medicine, Philadelphia, USA; 3 Psychiatry, West Virginia School of Osteopathic Medicine, Lewisburg, USA; 4 Psychiatry, West Virginia University School of Medicine, Martinsburg, USA; 5 Psychiatry, Philadelphia College of Osteopathic Medicine, Philadelphia, USA

**Keywords:** k2/spice, synthetic cannabis, synthetic cannabinoids, appetite, cb-1, appetite stimulation

## Abstract

This case report explores the use of K2/Spice (synthetic cannabinoids) in a patient as an appetite stimulant. The effects of synthetic cannabinoids range widely but are more commonly known to suppress appetite in the cannabinoid-naive. Our patient was not cannabinoid- naive and had used cannabis before. Rat models have demonstrated results similar to those in this patient, who had prior tetrahydrocannabinol (THC) exposure resulting in appetite stimulation rather than suppression when exposed to a synthetic cannabinoid. This is likely explained by other rat models that have shown long-term use of cannabis resulting in receptor downregulation of cannabinoid receptor type 1 (CB-1).

## Introduction

This report analyzes the use of K2/Spice in a patient for the purpose of appetite stimulation. The review of an article on Spice that included 50 cases and their listed adverse effects has excluded appetite stimulation as one of its uses [1]. There is also a high level of concern that Spice can induce psychosis in patients [2]. These concerns are the biggest roadblocks when it comes to the use of synthetic cannabinoids for therapeutic purposes like appetite stimulation [3]. Rat models have shown that previous exposure to cannabis can “unmask” the appetite-stimulating effects of Spice and could help with formulating ways to attenuate adverse effects in the therapeutic use of synthetic cannabinoids [4].

## Case presentation

A 40-year-old female presented to the emergency department (ED) with influenza. She was found to have hypokalemia with potassium of 3.3 mEq/L and moderate persistent asthma. Hypokalemia was managed with IV fluid hydration and 20-mL KCl. She received Tamiflu (Genentech, Inc., San Francisco, CA) 75 mg for five days, Toradol (Roche Holding AG, Basel, Switzerland) 15 mg IV, and ibuprofen 600 mg as supportive management for influenza. Moderate persistent asthma was treated with budesonide and formoterol fumarate via nebulizer. A consult was made with psychiatry because she admitted to an eating disorder and polysubstance abuse. She had been suffering from a longstanding lack of appetite since the age of 16 and perceived herself as too thin. She expressed a desire to gain weight. She was found to have a BMI of 15.1 in the ED and had been incredibly frustrated by the lack of appetite stimulation despite her prescribed use of olanzapine. During the interview, she mentioned with distress that her weight was down to 90 pounds. She complained of nausea when attempting to eat without the aid of cannabinoids. She informed us that she had had a poor appetite since the age of nine and admitted to experiencing abuse in her childhood. She had originally used cannabis for appetite stimulation daily. Five years ago, she had lost custody of her children to their father. She had had to undergo drug screens by the authorities after this and, as a result, she had switched to K2 since it was not detectable on drug screens. When she used K2, she would binge to the extent that she would often end up vomiting. The patient’s history of drug use included one pack of cigarettes daily, narcotics, and benzodiazepines, with multiple rehabilitations. Her most recent rehab had been three months ago. She was temporarily living with her mother but said she would have to move out as it was subsidized housing. During the interview, she described herself as an addict and homeless. There was no mention of addiction to cocaine during the interview, but she was found to be cocaine- positive on a urine drug screen. She was involved with Alcoholics Anonymous and Narcotics Anonymous.

The patient was not interested in going to rehab at the time we spoke to her. Our evaluation of the patient found that she did not meet the criteria for anorexia nervosa. The Diagnostic and Statistical Manual of Mental Disorders, Fifth Edition (DSM-5) requires that three criteria be met for a diagnosis of anorexia nervosa : (1) restriction of energy intake that leads to low body weight; (2) intense fear of gaining weight or becoming fat or persistent behavior that prevents weight gain; and (3) distorted perception of body weight and shape [1]. The patient met the first criteria with her BMI of 15.1 but failed to meet the other two. The patient was very motivated to gain weight and was exasperated that medicine was failing to improve her appetite. She was on olanzapine at the time of admission, which she claimed did not improve her appetite. There was no fear of gaining weight on her part and, in fact, she had a desperate desire to gain weight. The patient also did not meet criteria three as she perceived herself as underweight and found it grotesque. She similarly did not meet the criteria for bulimia nervosa because one of DSM-5's key criteria for the condition is inappropriate compensatory behavior to prevent weight gain [1]. Contrary to this, she was, in fact, doing everything she could to gain weight. Two days after our interview, the patient was discharged from the ED with the resolution of flu-like symptoms and hypokalemia as well as improvement of shortness of breath and cough from asthma. Her potassium at discharge was 3.7 mEq/L.

## Discussion

Most of the current literature on K2/Spice focuses on its negative effects with the most frequent side effects noted being tachycardia, hypertension, agitation, hallucinations, and confusion [2]. In general, K2/Spice is more adequately described as an umbrella that includes over 120 cannabimimetic compounds [2]. While normally described as “synthetic cannabis”, all but one of these compounds, HU-210, are structurally distinct from Delta-9 THC, the primary psychoactive compound in cannabis [2]. The response to synthetic cannabinoids is incredibly variable, with distinct differences evident in active compounds even within a single batch [2]. The prominence of adverse effects in the use of K2/Spice in comparison to THC is likely due to synthetic cannabinoids being full agonists at primarily CB-1 receptors while THC is only a partial agonist at both CB-1 and CB-2 receptors [3]. In view of this, it makes sense that much of the clinical evidence points to K2/Spice having the adverse effects of a high dose of cannabis. The difficulties of finding a therapeutic use for full CB-1 receptor agonists lie in their side effects of causing euphoria, dysphoria, memory disturbances, tolerance, dependence, withdrawal, and diminished psychomotor performance in addition to being able to stimulate appetite [4]. The patient in question is a long-time user of cannabis, which may explain her response to K2. A study in rats has found that the adverse effects of K2 can be attenuated by previous exposure to THC [5]. In the said experiment, rats were found to receive the more positive effects of the synthetic cannabinoid at low doses of synthetic cannabinoid JWH-018, having been previously exposed to THC [5]. Rat models have also already previously found that long-term use of THC results in the global downregulation of cannabinoid receptors [6]. The search for an ideal cannabinoid agonist continues with attempts to overcome the adverse side effects as well as the development of tolerance. Previous attempts at using CB-1 receptors such as the inverse agonist Rimonabant (Sanofi, Paris, France) have shown a 2.5-fold increase in psychiatric events in a meta-analysis, forcing it to be removed from the market [7]. Ways to circumvent the dependency of CB-1 receptor agonists are currently underway with methods such as neutral antagonists, peripherally restricted antagonists, and allosteric modulators [8]. This case is unique in that it is the first reported instance showing similar attenuation of adverse effects in a human model.

## Conclusions

Our patient's use of K2/Spice for appetite stimulation is consistent with results in rat models. We feel that this may not be a single odd case but one that can be used in finding other novel ways to attenuate the less desirable effects of synthetic cannabinoids.
